# Evolution of genomic structural variation and genomic architecture in the adaptive radiations of African cichlid fishes

**DOI:** 10.3389/fgene.2014.00163

**Published:** 2014-06-03

**Authors:** Shaohua Fan, Axel Meyer

**Affiliations:** Lehrstuhl für Zoologie und Evolutionsbiologie, Department of Biology, University of KonstanzKonstanz, Germany

**Keywords:** SNPs, insertions, deletions, inversions, adaptive radiation, structural variation

## Abstract

African cichlid fishes are an ideal system for studying explosive rates of speciation and the origin of diversity in adaptive radiation. Within the last few million years, more than 2000 species have evolved in the Great Lakes of East Africa, the largest adaptive radiation in vertebrates. These young species show spectacular diversity in their coloration, morphology and behavior. However, little is known about the genomic basis of this astonishing diversity. Recently, five African cichlid genomes were sequenced, including that of the Nile Tilapia (*Oreochromis niloticus*), a basal and only relatively moderately diversified lineage, and the genomes of four representative endemic species of the adaptive radiations, *Neolamprologus brichardi, Astatotilapia burtoni, Metriaclima zebra*, and *Pundamila nyererei*. Using the Tilapia genome as a reference genome, we generated a high-resolution genomic variation map, consisting of single nucleotide polymorphisms (SNPs), short insertions and deletions (indels), inversions and deletions. In total, around 18.8, 17.7, 17.0, and 17.0 million SNPs, 2.3, 2.2, 1.4, and 1.9 million indels, 262, 306, 162, and 154 inversions, and 3509, 2705, 2710, and 2634 deletions were inferred to have evolved in *N. brichardi, A. burtoni, P. nyererei*, and *M. zebra*, respectively. Many of these variations affected the annotated gene regions in the genome. Different patterns of genetic variation were detected during the adaptive radiation of African cichlid fishes. For SNPs, the highest rate of evolution was detected in the common ancestor of *N. brichardi, A. burtoni, P. nyererei*, and *M. zebra*. However, for the evolution of inversions and deletions, we found that the rates at the terminal taxa are substantially higher than the rates at the ancestral lineages. The high-resolution map provides an ideal opportunity to understand the genomic bases of the adaptive radiation of African cichlid fishes.

## Introduction

Cichlid fishes provide one of the most extreme examples for adaptive radiations in vertebrates (Kocher, 2004; Salzburger and Meyer, 2004; Kuraku and Meyer, 2008; Henning and Meyer, in press). More than 2000 species have evolved during the last few million years in the Great Lakes of East Africa (Meyer, 1993; Salzburger et al., 2005; Elmer et al., 2009). With >250 endemic species in Lake Tanganyika, >800 species in Lake Malawi and >500 species in Lake Victoria, these are the largest adaptive radiations in vertebrates (Meyer et al., 1990; Stiassny and Meyer, 1999; Kocher, 2004; Henning and Meyer, in press). Lake Tanganyika is maximally 7–8 million years old, and is the oldest of these lakes (Sturmbauer et al., 1994). Lake Malawi is younger at about 2–4 Million years and the current form of Lake Victoria is probably less than 100,000 years old. These young species, particularly of Lakes Malawi and Victoria are both extremely young, yet spectacularly diverse in morphology, coloration and behavior. Since most species of Lakes Malawi and Victoria can be hybridized in the laboratory for forward genetic studies, we referred to them to be “natural mutants” that can be used for studying genomic diversification by natural and sexual selection (Meyer, 1993; Meyer et al., 1993, 1995; Kuraku and Meyer, 2008; Henning and Meyer, in press). A large body of work, including transcriptome sequencing (Salzburger et al., 2008; Lee et al., 2010; Baldo et al., 2011; Gunter et al., 2013), BAC library construction (Lang et al., 2006), candidate gene sequencing (Terai et al., 2002, 2006; Hofmann et al., 2009; Fan et al., 2011), and microarrays (Gunter et al., 2011; Loh et al., 2013), has been conducted in an effort to study the molecular basis of the adaptive radiation of African cichlid fishes (reviewed by Fan et al., 2012).

Recently, the cichlid genome consortium sequenced five African cichlid genomes, including the Nile Tilapia (*Oreochromis niloticus*), *Neolamprologus brichardi* (endemic to Lake Tanganyika), *Astatotilapia burtoni* (lives in and around Lake Tanganyika), *Metriaclima zebra* (endemic to Lake Malawi), and *Pundamila nyererei* (endemic to Lake Victoria) (Brawand et al., submitted). The analysis of these five African cichlid genomes shows that their rapid evolution is associated not with one, but with multiple mechanisms, including an excess of gene duplications, transposable element expansions, fast evolution of conserved non-coding elements, and the evolution of novel micro RNAs (Brawand et al., submitted). However, genomic work on cichlid diversification has lacked so far a comprehensive comparative analysis of large-scale genomic variation. Ordered by size, genomic variation can take on the form of single nucleotide polymorphisms (SNPs), short insertions and deletions (indels), and larger structural variation (SVs, normally > 50 bp). Furthermore, SVs can be classified as insertions, inversions, deletions, duplications and translocations (Alkan et al., 2011a). The advent of next generation sequencing (NGS) technologies has revolutionized the study of genomic variation (Alkan et al., 2011a), as high-density maps were generated for various model systems. Such maps, reveal the presence of a wide spectrum of variation and are of important for gaining a deeper understanding of phenotypic diversification and speciation from a genomic perspective (Quinlan et al., 2010; Elmer and Meyer, 2011; Mills et al., 2011b; Zhan et al., 2011; Jones et al., 2012b; Feulner et al., 2013; Zichner et al., 2013). For example, recent studies have shown that adaptive evolution in three spine stickleback is associated with the reuse of standing variations, but also with SVs such as chromosomal inversion and deletions (Chan et al., 2010; Jones et al., 2012a,b; Feulner et al., 2013).

In this study, using the recently sequenced five African cichlid genomes, we investigated patterns of genomic variation that accompany the adaptive radiation of African cichlids. The aims of the present study were 3-fold: first, we characterized the prevalence and locations of the genomic variation in these five African cichlid genomes. Second, we analyzed the variation between these genomes in a phylogenetic context, which enabled us to gain a deeper understanding of questions such as: when did this variation originate? How do processes such as natural selection operate on different types of variation? Third, we inferred the potential functional impact of this variation using gene annotation information. These analyses of the genomic variation will not only enable us to assess the impact of the genomic differentiation on the functional portions of the genome, but also elucidate not only correlative changes, but also possibly causal structural changes as potential mechanisms for the rapid evolution of cichlid fishes.

## Materials and methods

### Data collection

The Broad Institute determined the five African cichlid genomes using Illumina technology. Briefly, one individual per species was sequenced using paired-end and mate-paired libraries. The raw reads of each species were assembled using ALLPATHS-LG pipeline (Gnerre et al., 2011). The Tilapia genome was further anchored into 22 linkage groups using linkage map information (Brawand et al., submitted). The information of the five African cichlid genomes is listed in the Supplementary Table 1. In this study, the raw sequencing data were downloaded from the NCBI SRA database (for accession numbers of the libraries see Supplementary Table 2).

### Data processing

The SRA files were converted to Fastq format using the fastq-dump (version 2.3.2) with the NCBI SRA toolkit. Sickle (https://github.com/najoshi/sickle) was used to remove the sequencing adaptors, to mask bases with quality score lower than 20, and to exclude reads less than 50 bp in length with parameters -q 20, -l 50.

We processed the reads from the paired-end and mate-paired libraries separately. The overlapping paired-end reads (insertion size: 180 bp, sequencing length: 100 bp) were first trimmed and we only kept the first and the last 50 bp in the first and second read using fastx_trimmer of the Fastx toolkit (version 0.0.13) (http://hannonlab.cshl.edu/fastx_toolkit/). For the mate-paired libraries (insertion size 3000 bp, sequencing length: 36 bp), we first mapped the raw reads to their corresponding genomes using BWA (version 0.7.3a-r367) (Li and Durbin, 2009) and excluded the read pairs that are facing each other, as these reads could be potential contaminations of paired-end reads in the mate-paired libraries. The remaining mate-paired reads were reverse-complemented, therefore the orientation of the mate pair reads are as same as the paired-end reads, to fit the requirements of the software in the downstream analyses.

The filtered paired-end and mate-paired reads from Tilapia, *N. bricharid, A. burtoni, P. nyererei*, and *M. zebra* genomes were mapped to the anchored Tilapia genome using Burrows-Wheeler Aligner (BWA) with the default parameters (version 0.7.3a-r367) (Li and Durbin, 2009). Although mapping short reads against a relative distantly related (around 4% sequence divergence in coding regions) outgroup can be a challenge for BWA, the reference genome is equidistant to *N. bricharid, A. burtoni, P. nyererei*, and *M. zebra*, thus would not bias the placement of the reads and not affect the downstream analyses. The raw mapping results were converted to BAM format and ambiguously mapped reads removed by requiring a mapping quality ≥ 20 using Samtools (version 0.1.19-44428cd) (Li et al., 2009). Duplicated read pairs were removed using the MarkDuplicates in the Picard toolkit (version 1.92) (http://picard.sourceforge.net/). The filtered bam files from the former steps were utilized for variation detection.

### SNPs and indels

SNPs and indels were genotyped using GATK (version 2.6.5) (McKenna et al., 2010; Depristo et al., 2011). Specifically, reads in the indel regions were realigned locally to minimize the number of the mismatching bases in each read; the raw SNP and indel callings were filtered with the parameters of phred-scaled quality score >30, depth of coverage between 6 and 5000, and the strand bias based on the phred-scaled *p*-value using Fisher's exact test <200. We intersected the coordinates of the SNPs and indels with the transposable element regions in the Tilapia genome using the intersectBed in the Bedtools version v2.17.0 (Quinlan and Hall, 2010). The SNPs and indels located in the transposable element regions were excluded in the further analyses. The effects of the SNPs and indels were estimated by the SNPEff version 3.3f based on the gene annotation information of the Tilapia genome. We classified SNPs and indels as intergenic, intronic, upstream (within 5 kb upstream of a gene), downstream (within 5 kb downstream of a gene) and exonic ones. The highest effect of a SNP or indel was selected using VariantAnnotator in the GATK toolkit (McKenna et al., 2010; Depristo et al., 2011). The presence and absence the SNPs and indels sites across different species was checked using the Seqmule pipeline (http://seqmule.usc.edu).

### Structural variation

SV was detected with Pindel (version 0.2.5a1) with default parameters. As quality control, we first detected SVs using the mapping result of Tilapia reads against the Tilapia genome. SVs then found in the Tilapia genome were considered to be potential assembly errors. Therefore, the SVs detected in the *N. brichardi, A. burtoni, P. nyererei*, and *M. zebra* genomes were filtered out if they overlapped with the SVs in the Tilapia genome.

For the *N. brichardi, A. burtoni, P. nyererei*, and *M. zebra* genomes, we first estimated the SVs based on the mapping results of the paired-end and mate-paired libraries separately and merged the results with the same species using mergeBed in the Bedtools version v2.17.0 (Quinlan and Hall, 2010).

The raw results from Pindel were converted to the VCF format using pindel2vcf in the Pindel package (Ye et al., 2009). All the SVs that overlapped with the transposable element regions in the Tilapia genome were excluded from further analyses. Genes overlapping with the SV regions were identified using intersectBed in the Bedtools version v2.17.0 (Quinlan and Hall, 2010). The enrichment of the gene ontology (GO) in the SV regions was examined using the Fisher's exact test (with FDR correction <0.05) in Blast2GO (Conesa et al., 2005; Gotz et al., 2008). We used Multovl version 1.2.98 with default parameters to check whether SVs overlaps across different species (Aszodi, 2012).

### Origin and rate of genetic variation

The origin of the genetic variation (e.g., SNPs, indels, inversions, and deletions) was analyzed in a phylogenetic context. We mapped the variation to the phylogeny from the African cichlid genome project, which was estimated using around 2.7 million 4-fold degenerate sites from the alignments of nine teleost genomes (zebrafish, fugu, tetraodon, stickleback, medaka, and the five African cichlid genomes). Based the maximum parsimony assumption, if a variation was shared by sister taxa, we assumed the structural variant had evolved in the common ancestor of these two species or sister lineages rather than having evolved independently in two species. The origin of variation shared by non-sister taxa is hard to determine using this method, as these variants could evolve independently in different species or due to the low sample size (one individual per species) used in this study. Therefore, we excluded these variants from the further rate analyses. To compare the rates of variation at different lineages, we first estimated the divergence time of the species used in this study. By assuming the cichlids in Lake Victoria (*P. nyererei*) and Lake Malawi (*M. zebra*) diverged from their most recent common ancestor on average 2.3 million years ago (Friedman et al., 2013), the divergence time for other lineages was estimated using a non-parametric approach (Sanderson, 1997) in the TreeEdit version v.1.0a10 (Rambaut and Charleston, 2002). Then, the rate of variation was calculated by dividing the number of lineage specific variation by the divergence time.

## Results

In total, 992 Gb raw sequence data were analyzed in this study (Supplementary Table 2). We achieved at least 20x coverage for all the species after filtering with the criteria mentioned before. In the following sections, we will focus on the genomic variation in 22 linkage groups, which make up about 71% of the whole Tilapia genome assembly.

**Figure 1:**
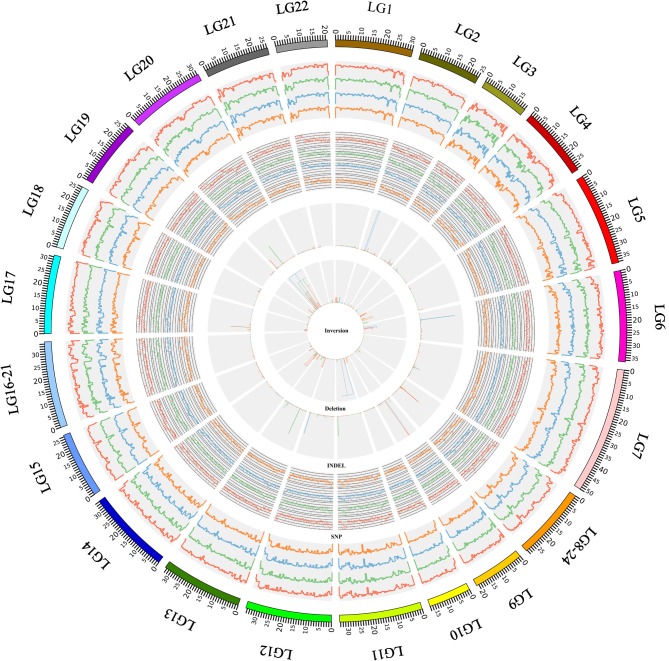
**The distribution of genomic variation in the African cichlid genomes**. This figure shows an overview of the SNPs, indels, inversions, and deletions that were identified in this study. The most outer ring showed the chromosomal ideogram in a clockwise orientation. The panels from outside to inside summarized the SNP density, indel density, and the location of the inversions and deletions, which were identified in the *N. brichardi* (in red), *A. burtoni* (in green), *P. nyererei* (in blue), and *M. zebra* (in orange) genomes. The SNP and indel densities were scanned using a 50 kb sliding window. The histograms in the inversion and deletion panels represent the position of the inversion and deletion and the widths of the histograms show the size of the variations. The results were visualized using Circos (Krzywinski et al., 2009).

Around 17 million SNPs were detected in the *N. brichardi, A. burtoni, P. nyererei*, and *M. zebra* genomes using the Tilapia genome as the reference genome (Table 1, Figure 1). Whereas most of the SNPs are located in the intergenic and intronic regions of the genomes (>70%), many SNPs (around 4%) are located the exonic regions of all four species (Table 1) and the ratio between non-synonymous and synonymous SNP is around 0.7 (Table 1). Of all 20 162 genes on the 22 linkage groups, we found around 77% of the genes with at least one non-synonymous SNP (data not shown). Except for the genes in the *N. brichardi* genome, the top 1% of the genes with highest proportion of the non-synonymous SNPs (normalized by the exon length) is significant enriched in the pathways related to immune response in the *A. burtoni, P. nyererei*, and *M. zebra* genomes.

**Table 1:** **Summary of the SNPs and indels in the African cichlid genomes**.

		***N. brichardi***	***A. burtoni***	***P. nyererei***	***M. zebra***
SNP	Synonymous (%)	440,813 (2.3)	410,635 (2.3)	411,584 (2.4)	405,220 (2.4)
	Non-synonymous (%)	311,551 (1.7)	289,739 (1.6)	290,001 (1.7)	291,171 (1.7)
	Upstream (%)	1,503,602 (8.0)	1,415,307 (8.0)	1,346,770 (7.9)	1,349,864 (8.0)
	Downstream (%)	2,940,995 (15.6)	2,784,038 (15.7)	2,659,723 (15.7)	2,664,669 (15.7)
	Intron (%)	7,693,416 (40.8)	7,232,387 (40.8)	6,962,386 (41.0)	6,958,755 (41.0)
	Intergenic (%)	5,955,045 (31.6)	5,577,008 (31.5)	5,319,811 (31.3)	5,303,501 (31.2)
	Total	18,845,422	17,709,114	16,990,275	16,973,180
Indel	Exon (%)	15,583 (0.7)	14,909 (0.7)	9912 (0.7)	13,663 (0.7)
	Upstream (%)	183,740 (7.9)	174,367 (7.9)	113,395 (7.9)	147,365 (7.9)
	Downstream (%)	413,967 (17.9)	394,421 (18.0)	259,539 (18.0)	337,612 (18.0)
	Intron (%)	1,007,681 (43.5)	959,217 (43.7)	629,137 (43.7)	819,930 (43.8)
	Intergenic (%)	696,342 (30.0)	651,827 (29.7)	428,708 (29.7)	554,392 (29.6)
	Total	2,317,313	2,194,741	1,440,691	1,872,962

The intergenic, intronic, up- and downstream, and exonic regions were based on the annotation of the Tilapia genome.

The number of indels in these data sets ranges from 1,440,691 to 2,317,313 in these four species (Table 1). Proportionally, indels occur as frequently as SNPs in the intronic, intergenic, upstream and downstream regions, but are less common in the exonic regions (<1% vs. >4%, Table 1). Based on the gene annotation of the Tilapia genome, some of the exonic indels may have deleterious effects, such as causing frameshifts or the loss of start and stop codons. In comparison to the genome-wide indels, the exonic indels are enriched for sizes divisible by three, which indicates that those indel events may affect a whole codon (Figure 3).

Using paired-end and mate-paired data, Pindel identified 621, 683, 371, and 389 inversions in the *N. brichardi, A. burtoni, P. nyererei*, and *M. zebra* genomes, respectively, (Table 2). Normalized by the length of linkage groups, we did not detect any linkage group that has a significantly high number of inversions than any other in the *N. brichardi, A. burtoni, P. nyererei*, and *M. zebra* genomes (generalized ESD test, *p*-value > 0.05) (Rosner, 1983). The average length of the inverted region ranges from 98,025 to 214,075 bp in the species compared (Table 2). The longest inversion, 24,423,751 bp, was located on LG20 of the *P. nyererei* genome and involves 853 genes. In total, we found that 262, 306, 162, and 154 genes overlapped with the inverted regions in the *N. brichardi, A. burtoni, P. nyererei*, and *M. zebra* genomes (Table 2). However, no GO terms are significantly enriched in the inverted regions.

**Table 2:** **Summary of the inversions and deletions in the African cichlid genomes**.

		***N. brichardi***	***A. burtoni***	***P. nyererei***	***M. zebra***
Inversion	Number	621	683	371	389
	Longest (bp)	1,13,74,460	1,90,74,784	2,44,23,750	1,43,18,936
	Shortest (bp)	51	51	51	51
	Average length (bp)	98,025	58,793	214,075	161,034
	Number of genes affected	262	306	162	154
Deletion	Number	15,833	10,695	9840	10,070
	Longest (bp)	9,82,119	6,34,424	24,45,312	7,90,935
	Shortest (bp)	50	50	50	50
	Average length (bp)	561	795	981	646
	Number of genes affected	3509	2705	2710	2634

The number of the genes affected indicates the number of the genes overlaps with in the inversion and deletion regions.

In addition to SNPs, indels and inversions, we also assessed long deletions (>50 bp) in the African cichlid genomes. 15,833, 10,695, 9,840, and 10,070 long deletions were identified in the *N. brichardi, A. burtoni, P. nyererei*, and *M. zebra* genomes, respectively, (Table 1). However, no linkage group harbors a significantly higher number of long deletions after normalizing by the length of linkage group (generalized ESD test, *p*-value > 0.05) (Rosner, 1983). The *N. brichardi* genome has the greatest number of deletions; however, their average length in the *P. nyererei* genome is larger than in the other species (Table 2). Investigating this further with the annotated gene regions, we found that the genes affected by the deleted regions were significantly associated with the GO terms “binding,” “cell adhesion,” and “biological adhesion” (FDR corrected *p*-value < 1E-4).

**Figure 2:**
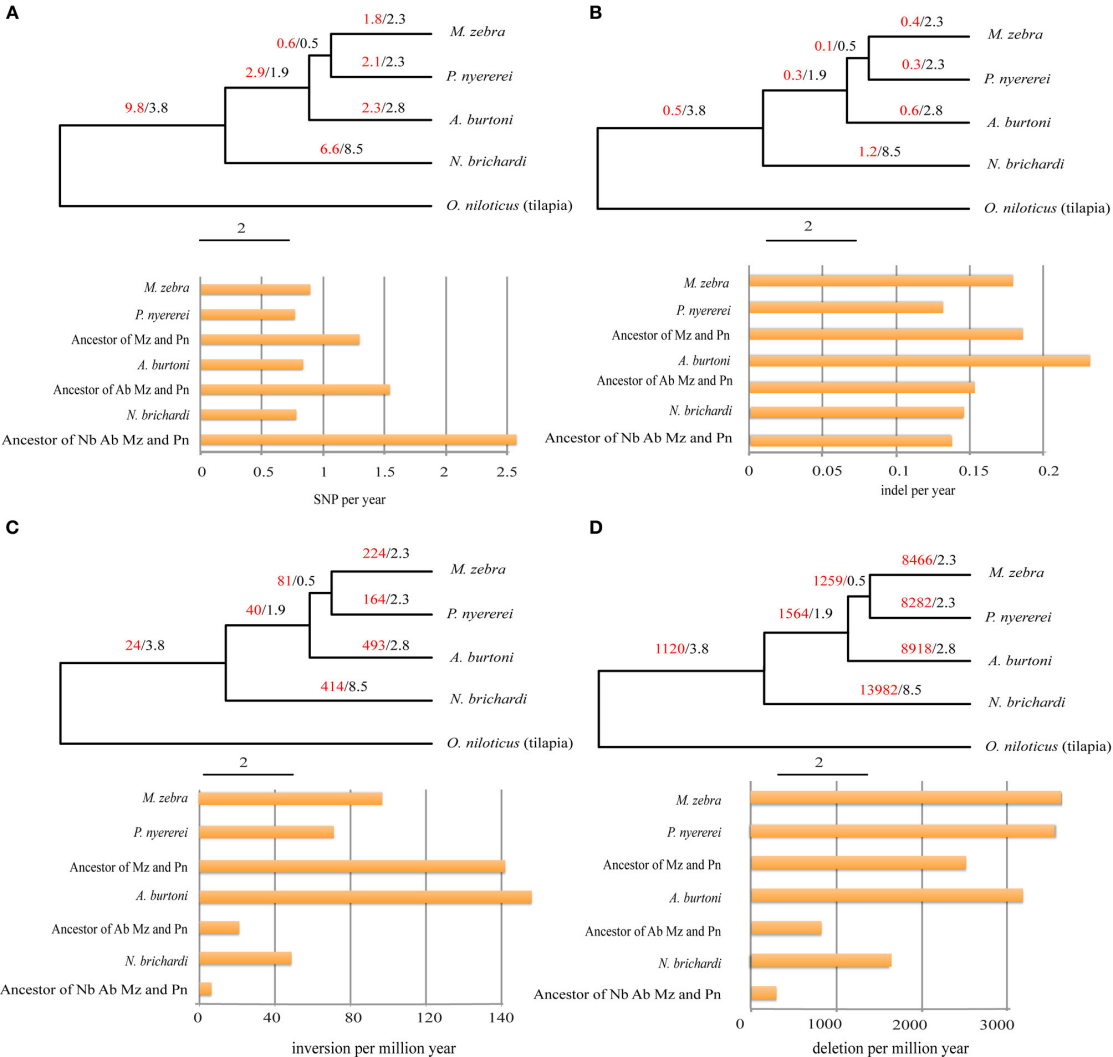
**The evolutionary patterns of SNPs, indels, inversions, and deletion during the adaptive radiation of African cichlids**. The phylogeny was adopted from the cichlid genome paper (Brawand et al., submitted). The evolution of SNP, indel, inversion, and deletion were shown in **(A–D)**. The numbers before and after slash are the number of the lineage specific variation and the divergence time (million years). The numbers of the SNPs and indels on the phylogeny were scaled by millions. The numbers of the inversions and deletion were not scaled by millions. The histogram shows the number of the variations normalized by the corresponding divergence time. Nb, Ab, Pn, and Mz indicate *Neolamprologus brichardi, Astatotilapia burtoni, Pundamila nyererei*, and *Metriaclima zebra*.

Different patterns of structural genetic variation were detected during the divergence of the African cichlids (Figure 2). Large numbers of SNPs (9.8 million) can be traced back to the common ancestor of *N. brichardi, A. burtoni, P. nyererei*, and *M. zebra* and indicate in a substantially higher rate of SNP evolution (from 1.7 TO 3.3-fold changes) in comparison to other lineages (Figure 2A) at the basis of the radiation of African cichlids. However, we did not detect similar patterns in the evolution of inversions and deletions. The rates of inversions and deletions in the common ancestor of these four species are the slowest rate in comparison to other lineages, especially in comparison to the haplochromine lineages (Figures 2C,D). The rate of indels evolved uniformly in all lineages (Figure 2B).

## Discussion

The genome sequence data collected by the cichlid genome consortium provides an opportunity for the study of genome evolution of African cichlid fishes. Using these whole genome sequencing data, we generated a high-resolution map of genomic variation in African cichlid fishes (Figure 1). The resolution of most of this genetic variation down to the nucleotide level enabled us to conduct this initial investigation of their potential impacts to the adaptive radiation of African cichlid fishes.

SNPs are the most common form of genetic variation in the cichlid genomes. On average, we found around 17 million SNPs in the genomes of *N. brichardi, A. burtoni, P. nyererei*, and *M. zebra* using the Tilapia genome as the reference genome. Although the relative low number in comparison to the SNPs in the other regions, many non-synonymous SNPs evolved during the adaptive radiation of African cichlids (Table 1). We found that the genes involved in immune pathways, for example, Interleukin-15, major histocompatibility complex II (MHC II), and C-C motif chemokine 19 have an excessive number of non-synonymous SNPs. Immune genes are commonly characterized by signature of positive selection during speciation process (Jansa et al., 2003; Nielsen et al., 2005; Jiggins and Kim, 2007; Jones et al., 2012a). The selection can be strongly intensified during the colonization of new habitats as new pathogens may induce primary challenges to host immune system (Matthews et al., 2010; Jones et al., 2012a).

The extensive amount of SNP sites that is shared across the genomes of *N. brichardi, A. burtoni, P. nyererei*, and *M. zebra* could be the result of two scenarios. First, these may be deeply shared polymorphisms, which originated in the common ancestor of *N. brichardi, A. burtoni, P. nyererei*, and *M. zebra* and are still present in these species. Previous study has shown that genetic polymorphisms in the endemic cichlids of Lake Victoria can be traced back to the ancestors of the modern haplochromine lineages (Elmer et al., 2009). Furthermore, genotyping a set of 280 SNPs in ~160 African cichlid species, Loh and coauthors found that around 50% of these loci are polymorphic in the cichlid lineages across East Africa (Loh et al., 2013). Second, it may be that ongoing but occasional genetic interchange among lacustrine cichlids from different great lakes is facilitated by riverine cichlid species as “transporters” (Verheyen et al., 2003; Loh et al., 2013). Such ongoing gene flow among cichlid species could transfer beneficial alleles among species, thus promoting the rapid evolution of African cichlids because these pre-existing genetic variations may have already been tested by natural selection in similar environments (e.g., in adaptation to lacustrine environments).

Comparing the rate of SNP evolution during the evolution of the adaptive radiations of African cichlids, a higher rate of SNP evolution was observed in the lineage of the common ancestor of all four representatives of the three East African cichlid radiations: *N. brichardi, A. burtoni, P. nyererei*, and *M. zebra*, compared to the terminal branches (Figure 2A). This could be the result of relaxed purifying selection or positive selection for adaptation to the novel niches in the newly colonized lakes, compared to an ancestral riverine environment that would have been inhabited by the ancestral lineages to the endemic radiations. Environmental factors often lead to chanced selection pressures that either eliminates or weakens the purified selection that might have dominated in previous habitats (Lahti et al., 2009) or increases the frequency of the beneficial alleles by positive natural selection (Nielsen et al., 2007).

**Figure 3:**
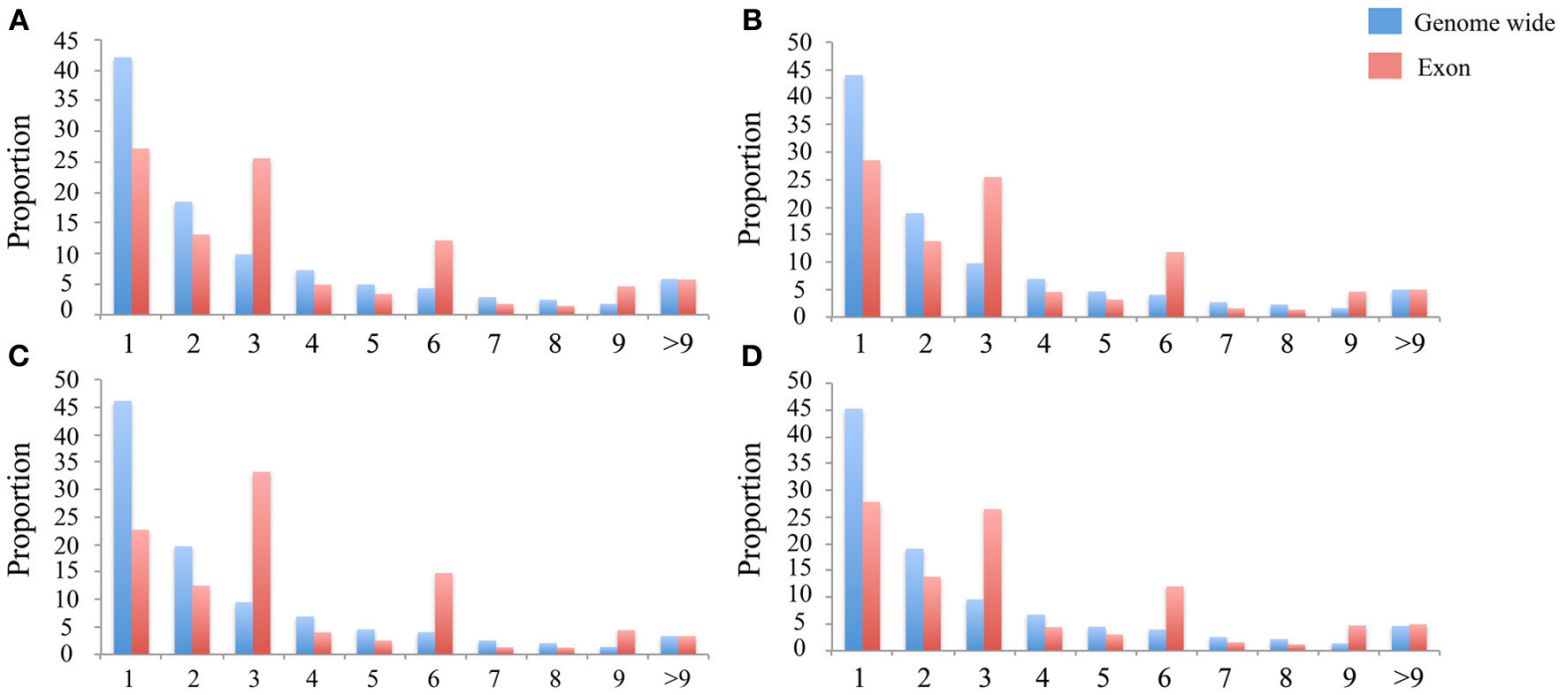
**Indel length distribution**. The Y-axis is the proportion of the indels in the exon region (Red) and genome wide (Blue). The X-axis is binned by the length of the indel. **(A–D)** present in the indel length distribution in the *N. brichardi, A. burtoni, P. nyererei*, and *M. zebra* genomes.

In comparison to SNPs, we found much fewer indels in exons (Table 1) and if there, the length is highly likely to be divisible by three, obviously to keep the reading frame intact (Figure 3). This was also reported for the genomes of cattle (Zhan et al., 2011), human (Mills et al., 2011a), and sticklebacks (Feulner et al., 2013). The rare presence of the indels in the exonic regions reflects the strong purify selection against this form of variation, which potentially disrupts protein coding. Based on the gene annotation of the Tilapia genome, some of those exonic indels may have deleterious effects, which needs further validation in the future. However, we cannot rule out that some predicted effects of the exonic indels resulted from an incorrect gene model in the Nextgen genomes (Alkan et al., 2011b) or from sequencing errors.

In contrast to the SNP evolution, the rates of SV in the haplochromine lineages are higher than in the ancestral lineages (Figures 2C,D). A large body of research has examined the role of genomic structure variation (including inversions and deletions) in local adaptation and speciation (Noor et al., 2001; Kirkpatrick and Barton, 2006; Feder and Nosil, 2009; Chan et al., 2010; Jones et al., 2012b; Feder et al., 2013). For example, multiple lines of evidence show that loci involved in local adaptation (Coluzzi et al., 2002; Anderson et al., 2005; Kirkpatrick and Barton, 2006; Lowry and Willis, 2010) and pre- or postzygotic isolations were mapped to inverted regions (Noor et al., 2001; Rieseberg, 2001; Ayala et al., 2013). The evolutionary importance of inversions lies in their ability to suppress recombination when they are heterozygous between population or species (Sturtevant, 1917). Therefore, co-adapted alleles may be embedded in the inverted regions and will not be eroded by introgression and recombination (Dobzhansky, 1947, 1970). Besides, structural variation can also affect the gene expression if the breakpoints of SVs overlap with regulatory regions (Wesley and Eanes, 1994; Chan et al., 2010; Harewood et al., 2012). For example, repeated deletions of the enhancer of the Pitx1 gene changed the gene expression patterns and is responsible for the repeated and independent loss of the pelvic fin in the freshwater stickleback populations (Chan et al., 2010). Our analyses of structural variation of five East Africa cichlid genomes provides a starting point for further investigations of the impact of structural variation during the adaptive radiation of African cichlid fishes. Especially, given the ongoing gene flow between cichlids from different lakes (Ruber et al., 2001; Verheyen et al., 2003; Elmer et al., 2009; Joyce et al., 2011; Loh et al., 2013), the lineage specific SV (Figures 2C,D) would be highly informative in understanding the processes and genomic consequences of speciation-with-gene-flow.

Our analyses provide a first glance at the genomic variation of genomes of cichlid fish of adaptive radiation of East Africa. Although only one individual per species was sampled so far, we detected a large amount of shared structural variation across the cichlid genomes of these closely related adaptive radiations, indicating that also this type of genomic variation is a form of shared ancestral variation that is maintained across species and lineages. Much of the structural variation of these genomes is located in functionally important regions of genes (e.g., exonic or regulatory regions). One might be tempted to speculate that also this type of variation might have contributed to both the local adaptation and speciation of cichlids—but this need further investigation through functional essays of candidate genes and structural variants, such as CRISPR-Cas (Kratochwil and Meyer, unpublished data). One next step is to evaluate the functional impacts of these regions during different waves of repeated adaptive radiations by systematically investigating the variability and selection pressures of these regions through population genomic analyses in representatives of the parallel-evolved species flocks of African cichlids.

### Conflict of interest statement

The authors declare that the research was conducted in the absence of any commercial or financial relationships that could be construed as a potential conflict of interest.
