# Supplementary material for: Evolution of genomic structural variation and genomic architecture in the adaptive radiations of African cichlid fishes
Source: Front Genet. 2014 Jun 3;5:163. doi: 10.3389/fgene.2014.00163 (PMC4042683; doi:10.3389/fgene.2014.00163)
Supplement: Supplementary file 1 [file DataSheet1.DOCX]

Supplementary Table 1. The information of the five African cichlid genome assemblies.

| Species | Genome size （bp）* | No. Scaffolds | Scaffold N50 (Mb)+ | Gene number |
| --- | --- | --- | --- | --- |
| *O. niloticus*^§^ | 927,679,487 | 5677 | 27 | 24,559 |
| *N. brichardi* | 847,893,845 | 9098 | 4.4 | 20,119 |
| *A. burtoni* | 831,411,547 | 8001 | 1.2 | 23,436 |
| *P. nyererei* | 830,133,247 | 7236 | 2.5 | 20,611 |
| *M. zebra* | 848,776,495 | 3075 | 3.7 | 21,673 |

*^§^* We used the Tilapia genome assembly

* The genome is the number of the base pairs in the assembled genomes.

^+^ N50 is a measure of the continuity of the genome assembly. It is a collection of the scaffolds that of length or longer than the half of the bases in an assembly.

Supplementary Table 2. The sequencing data used in this study. The accession number is originated from the NCBI SRA database.

| Species | Accession number | Library type | Insertion size (bp) | Number of bases (Gb) |
| --- | --- | --- | --- | --- |
| *Oreochromis niloticus* | SRR071619 | Paired-end | 180 | 6 |
|  | SRR071610 | Paired-end | 180 | 6,5 |
|  | SRR071605 | Paired-end | 180 | 6,8 |
|  | SRR071601 | Paired-end | 180 | 6,3 |
|  | SRR071594 | Paired-end | 180 | 6,5 |
|  | SRR071593 | Paired-end | 180 | 6,8 |
|  | SRR071589 | Paired-end | 180 | 6,1 |
|  | SRR071599 | Mate-paired | 3000 | 27.9 |
|  | SRR071612 | Mate-paired | 3000 | 27.8 |
|  | SRR071614 | Mate-paired | 3000 | 27.8 |
|  | SRR071603 | Mate-paired | 3000 | 28.4 |
|  | SRR071597 | Mate-paired | 3000 | 28.3 |
|  | SRR071591 | Mate-paired | 3000 | 28.1 |
|  | SRR071588 | Mate-paired | 3000 | 28 |
|  | SRR071608 | Mate-paired | 3000 | 2.7 |
|  | SRR071592 | Mate-paired | 3000 | 2.8 |
|  | SRR071587 | Mate-paired | 3000 | 2.8 |
|  | SRR071613 | Mate-paired | 3000 | 2.7 |
| *N. brichardi* | SRR077327 | Paired-end | 180 | 20 |
|  | SRR077329 | Paired-end | 180 | 19.7 |
|  | SRR077332 | Paired-end | 180 | 20.3 |
|  | SRR077340 | Paired-end | 180 | 19.7 |
|  | SRR077336 | Mate-paired | 3000 | 6.4 |
|  | SRR077339 | Mate-paired | 3000 | 6.5 |
|  | SRR077334 | Mate-paired | 3000 | 5.5 |
|  | SRR077341 | Mate-paired | 3000 | 5.7 |
|  | SRR077330 | Mate-paired | 3000 | 19.3 |
|  | SRR077333 | Mate-paired | 3000 | 19.9 |
|  | SRR077346 | Mate-paired | 3000 | 19.3 |
|  | SRR077328 | Mate-paired | 3000 | 15.3 |
| *A. burtoni* | SRR077270 | Paired-end | 180 | 18.5 |
|  | SRR077278 | Paired-end | 180 | 18.3 |
|  | SRR077279 | Paired-end | 180 | 18.3 |
|  | SRR077283 | Paired-end | 180 | 18 |
|  | SRR077277 | Mate-paired | 3000 | 10 |
|  | SRR077276 | Mate-paired | 3000 | 16.2 |
|  | SRR077274 | Mate-paired | 3000 | 12.9 |
|  | SRR077268 | Mate-paired | 3000 | 6 |
|  | SRR077271 | Mate-paired | 3000 | 6.2 |
|  | SRR077266 | Mate-paired | 3000 | 7.5 |
|  | SRR077281 | Mate-paired | 3000 | 7.7 |
|  | SRR077264 | Mate-paired | 3000 | 16.4 |
|  | SRR077262 | Mate-paired | 3000 | 4.9 |
|  | SRR077275 | Mate-paired | 3000 | 4.8 |
| *M. zebra* | [SRR077286](http://trace.ncbi.nlm.nih.gov/Traces/sra/sra.cgi?cmd=viewer&m=data&s=viewer&run=SRR077286) | Paired-end | 180 | 7.6 |
|  | [SRR077287](http://trace.ncbi.nlm.nih.gov/Traces/sra/sra.cgi?cmd=viewer&m=data&s=viewer&run=SRR077287) | Paired-end | 180 | 7.3 |
|  | [SRR077290](http://trace.ncbi.nlm.nih.gov/Traces/sra/sra.cgi?cmd=viewer&m=data&s=viewer&run=SRR077290) | Paired-end | 180 | 7.6 |
|  | [SRR077292](http://trace.ncbi.nlm.nih.gov/Traces/sra/sra.cgi?cmd=viewer&m=data&s=viewer&run=SRR077292) | Paired-end | 180 | 7.6 |
|  | [SRR077298](http://trace.ncbi.nlm.nih.gov/Traces/sra/sra.cgi?cmd=viewer&m=data&s=viewer&run=SRR077298) | Paired-end | 180 | 7.5 |
|  | [SRR077300](http://trace.ncbi.nlm.nih.gov/Traces/sra/sra.cgi?cmd=viewer&m=data&s=viewer&run=SRR077300) | Paired-end | 180 | 7.6 |
|  | [SRR077301](http://trace.ncbi.nlm.nih.gov/Traces/sra/sra.cgi?cmd=viewer&m=data&s=viewer&run=SRR077301) | Paired-end | 180 | 7.5 |
|  | [SRR077302](http://trace.ncbi.nlm.nih.gov/Traces/sra/sra.cgi?cmd=viewer&m=data&s=viewer&run=SRR077302) | Paired-end | 180 | 7.6 |
|  | [SRR077299](http://trace.ncbi.nlm.nih.gov/Traces/sra/sra.cgi?cmd=viewer&m=data&s=viewer&run=SRR077299) | Mate-paired | 3000 | 1.4 |
|  | [SRR077297](http://trace.ncbi.nlm.nih.gov/Traces/sra/sra.cgi?cmd=viewer&m=data&s=viewer&run=SRR077297) | Mate-paired | 3000 | 1.6 |
|  | [SRR077296](http://trace.ncbi.nlm.nih.gov/Traces/sra/sra.cgi?cmd=viewer&m=data&s=viewer&run=SRR077296) | Mate-paired | 3000 | 3 |
|  | [SRR077295](http://trace.ncbi.nlm.nih.gov/Traces/sra/sra.cgi?cmd=viewer&m=data&s=viewer&run=SRR077295) | Mate-paired | 3000 | 22 |
|  | [SRR077293](http://trace.ncbi.nlm.nih.gov/Traces/sra/sra.cgi?cmd=viewer&m=data&s=viewer&run=SRR077293) | Mate-paired | 3000 | 2.4 |
|  | SRR082783 | Mate-paired | 3000 | 17.3 |
|  | SRR082785 | Mate-paired | 3000 | 16.2 |
|  | SRR082791 | Mate-paired | 3000 | 13.3 |
|  | [SRR077288](http://trace.ncbi.nlm.nih.gov/Traces/sra/sra.cgi?cmd=viewer&m=data&s=viewer&run=SRR077288) | Mate-paired | 3000 | 2.3 |
|  | SRR077291 | Mate-paired | 3000 | 24.6 |
|  | SRR077294 | Mate-paired | 3000 | 23.9 |
| *P. nyererei* | SRR082778 | Paired-end | 180 | 21.2 |
|  | SRR082779 | Paired-end | 180 | 22 |
|  | SRR082781 | Paired-end | 180 | 21.3 |
|  | SRR082782 | Paired-end | 180 | 21.5 |
|  | SRR082797 | Mate-paired | 3000 | 1.7 |
|  | [SRR082794](http://trace.ncbi.nlm.nih.gov/Traces/sra/sra.cgi?cmd=viewer&m=data&s=viewer&run=SRR082794) | Mate-paired | 3000 | 3.2 |
|  | [SRR082792](http://trace.ncbi.nlm.nih.gov/Traces/sra/sra.cgi?cmd=viewer&m=data&s=viewer&run=SRR082792) | Mate-paired | 3000 | 3.2 |
|  | [SRR082791](http://trace.ncbi.nlm.nih.gov/Traces/sra/sra.cgi?cmd=viewer&m=data&s=viewer&run=SRR082791) | Mate-paired | 3000 | 13.3 |
|  | [SRR082789](http://trace.ncbi.nlm.nih.gov/Traces/sra/sra.cgi?cmd=viewer&m=data&s=viewer&run=SRR082789) | Mate-paired | 3000 | 2.3 |
|  | [SRR082788](http://trace.ncbi.nlm.nih.gov/Traces/sra/sra.cgi?cmd=viewer&m=data&s=viewer&run=SRR082788) | Mate-paired | 3000 | 1.9 |
|  | [SRR082786](http://trace.ncbi.nlm.nih.gov/Traces/sra/sra.cgi?cmd=viewer&m=data&s=viewer&run=SRR082786) | Mate-paired | 3000 | 3.5 |
|  | [SRR082785](http://trace.ncbi.nlm.nih.gov/Traces/sra/sra.cgi?cmd=viewer&m=data&s=viewer&run=SRR082785) | Mate-paired | 3000 | 16.2 |
|  | [SRR082783](http://trace.ncbi.nlm.nih.gov/Traces/sra/sra.cgi?cmd=viewer&m=data&s=viewer&run=SRR082783) | Mate-paired | 3000 | 17.3 |
|  | [SRR082780](http://trace.ncbi.nlm.nih.gov/Traces/sra/sra.cgi?cmd=viewer&m=data&s=viewer&run=SRR082780) | Mate-paired | 3000 | 1.9 |
|  | SRR077330 | Mate-paired | 3000 | 19.3 |
|  | SRR077333 | Mate-paired | 3000 | 19.9 |
|  | SRR077346 | Mate-paired | 3000 | 18.3 |
